# Transboundary Water Cooperation in the Post-Cold War Era: Spatial Patterns and the Role of Proximity

**DOI:** 10.3390/ijerph19031503

**Published:** 2022-01-28

**Authors:** Ziming Yan, Xiaojuan Qiu, Debin Du, Seamus Grimes

**Affiliations:** 1 Institute for Global Innovation and Development, East China Normal University, Shanghai 200062, China; 2 John. F. Kennedy School of Government, Harvard University, Cambridge, MA 02138, USA; 3 School of Politics and International Relations, East China Normal University, Shanghai 200062, China; x.qiu@uva.nl; 4 Amsterdam Institute for Social Science Research (AISSR), University of Amsterdam, 1001 NE Amsterdam, The Netherlands; 5 Whitaker Institute for Innovation and Societal Change, National University of Ireland, H91 TK33 Galway, Ireland; seamus.grimes@nuigalway.ie

**Keywords:** transboundary water cooperation, Post-Cold War era, social network analysis, QAP analysis, proximity

## Abstract

Transboundary water cooperation (TWC) is an important theme of international cooperation. We conducted macro-level research on TWC from the perspective of inter-country relations and constructed a theoretical framework in which multidimensional proximity influences the formation of global TWC. We explained how multidimensional proximity and the constituent elements comprehensively influence the cooperative willingness and ability of actors, which directly drive the generation of global TWC. During the empirical research phase, we constructed the TWC frequency and intensity networks based on historical TWC events data from 1992 to 2013. By using social network analysis and QAP regression analysis, the spatial structure and proximity effect of water cooperation linkages are examined. It can be found that: (1) the reconstruction of territorial space on the eve of the end of the Cold War led to the peak of water cooperation events in 1992. The overall scale of events in the Post-Cold War era was relatively high and fluctuated steadily. (2) Water cooperation linkages have distinct spatial heterogeneity and are concentrated in the Eurasian and the African continents. Water cooperation is sensitive to geographical distance, and high-intensity water cooperation linkages exist in only a few areas. (3) China, Egypt, Germany, the United States, and Russia have prominent positions in the network. The United States, Japan, and other extra-regional powers actively participated in TWC in the Eastern Hemisphere. (4) The regression results show that geographical, economic, organizational, and colonial proximity significantly affect the intensity of water cooperation among countries.

## 1. Introduction

Transboundary water is an important resource and a natural link that maintains relations between countries in the basin; this is also related to regional economic and social progress, world peace and stability, and the rapid development of human civilization. As of 2018, there are 310 international river basins in the world, shared by 150 countries, which cover 47.1% of the world’s land surface and have 52% of the world’s population residing within their boundaries [1]. However, shared water can indeed lead to regional tensions, threats, and even localized violence [2], and the unsustainable use of freshwater resources worldwide creates enormous challenges for human societies [3,4]. The excessive consumption of water resources in human production and life and the variation in water volume caused by climate change make international river basins face a great risk of conflict, and transboundary water resources are increasingly becoming the source of inter-country violent conflicts [5].

In 2014, the IPCC’s (Intergovernmental Panel on Climate Change) Fifth Assessment Report made a serious estimate of the risks for global freshwater resources caused by climate change. It emphasizes that the risks for freshwater related to climate change and extreme events will increase significantly under the scenario of global warming of 1.5 °C [6]. As environmental changes will eventually lead to an increase in the economic and political value of water resources, this will increase the possibility of disputes between countries around transboundary water resources, and even the risk of military conflicts [7]. Furthermore, international river basins extend beyond national jurisdictions and their policy-making structures, making effective policy responses to them more difficult and prone to failure [4]. In the face of this dilemma, many basin countries facing water stress urgently call for international collective action to strengthen the rational control and effective governance of transboundary water resources. In the context of the in-depth development of world multi-polarization and economic globalization, and the unprecedented deepening of interdependence among countries, actively developing transboundary water cooperation (TWC) has become an inevitable choice for basin countries to enhance mutual trust. Therefore, we focus our research on the spatial pattern and generation mechanism of TWC between countries, which could substantially further our understanding of TWC issues.

This research may contribute to the literature in three ways. First, we applied the social network analysis method to TWC on the global scale, and quantitatively evaluated the status of the countries in the TWC networks and the connections between countries. Additionally, we visualized the networks geographically to better reveal the spatial pattern of TWC. Second, with the help of the multidimensional proximity framework, we studied whether the specific four relationships between countries (geographical, economic, organizational, and colonial proximities) have an impact on the intensity of TWC among countries. Third, we proposed a model that proximities further affect the willingness and ability of actors to cooperate, and ultimately leads to the emergence of the TWC intensity network pattern among countries. Which also extends the previous analysis of the TWC generation mechanism between countries. This also extends the previous analysis of the TWC generation mechanism between countries. Besides that, we have also expanded the current TWC events database to 2013. This is helpful for further research on the progress of TWC.

The research is structured as follows. Section 2 presents the literature review and the theoretical framework in this article. Section 3 introduces the research areas, the data, and research methods. Our main findings and discussion are reported in Section 4, and the final section offers conclusions and future research directions.

## 2. Literature Review and Theoretical Framework

### 2.1. Literature Review

#### 2.1.1. Transboundary Water Cooperation

Various organizations have provided definitions of TWC. The UN-Water [8] considers TWC to be an “arrangement” established between transboundary basin countries, which may include bilateral or multilateral treaties or other formal arrangements. The European Union [9] believes that TWC and diplomatic issues are closely linked, aiming to urge countries to reach an agreement on the distribution and management of international shared water resources, and promote broader regional cooperation. The International Centre for Water Resources and Global Change [10] pointed out that although there is no singular definition of TWC, it can be regarded as a mutually beneficial exchange of two or more parties instead of competing for the same water resources. In academia, some scholars pointed out that water cooperation based on the signing of treaties is more effective. For example, Brochmann [11] and Dinar [12] emphasized the dominance of water treaties in TWC. Kistin [13] called on the academic community to go beyond the notion of cooperation as treaties and emphasized the important role of state and non-state actors. In general, there is currently no unified definition of TWC.

In this study, TWC, as the research object of the article, we believe that it should have four basic connotations. First, its essence involves the exchanges or mutual relations between multiple international political actors in the international community. Second, the actors include state actors and non-state actors, among which state actors occupy a dominant position. Third, the exchange or mutual relationship between actors is a cooperative relationship. Fourth, the object of cooperation is transboundary water resources, including two spatial forms that flow across national borders and form national borders by themselves. Therefore, we define TWC as “the actions of varying degrees of coordination, joint and mutual support between state actors, or between state and non-state actors, to ensure the realization of transboundary water resources development needs or related interest goals”. In this article, unless otherwise specified, TWC between state actors will be our focus for discussion.

Compared with attempts to define the concept, there has been abundant empirical research on TWC. The existing studies are mainly distributed in the fields of political science and geography, and there are some connections and differences in paradigms and research methods. From the perspective of the research paradigm, political scholars dominate the discussion on this topic. In particular, international relations scholars analyze regional water cooperation cases from the perspectives of liberalism, functionalism, constructivism, and institutionalism [14,15,16,17,18,19], in order to clarify the broader mechanism of international cooperation behind them. Compared with political scholars, geographers pay more attention to the response of TWC under climate change and the ecological challenges [20,21] that river basins may face, and they also emphasize the geographical complexity [22,23] of water cooperation in the process. Some scholars conducted research from the perspective of water supply and demand, and pointed out that under the pressure of freshwater resources, the sustainable development of transboundary basins and regional water cooperation are facing great uncertainty [12,24,25,26]. Although there are differences in research paradigms here, geographers generally agree that a broader political and historical background needs to be fully considered in TWC research [27].

From the perspective of research methods, most of the current TWC studies focus on individual international freshwater basins and emphasize policy options for solving the challenges of the region, such as case studies from the Mekong [28,29,30], Indus [31,32], Nile [33,34], or La Plata [35] River Basin. Some scholars have developed concepts and research methods to evaluate TWC and explore its driving forces, such as integrated water resources management (IWRM) [36,37], water diplomacy [38,39,40], and water-energy-food nexus [41,42,43], which deepens our understanding of TWC from the perspective of social science research. Among them, the Transboundary Freshwater Dispute Database (TFDD) project developed by Wolf et al. [44,45] has provided reliable spatial data and events data for quantitative assessment of global risk basins and water cooperation and conflicts, leading the trend of quantitative research in this field. By applying different research perspectives to water events, the basins with the potential for political stresses or conflicting interests on a global scale have been identified, trends in hydropolitics of transboundary basins have been discussed, and the most concerning areas of water cooperation have been confirmed [46,47,48]. Nevertheless, there have been three characteristics in this field for a long time: the paradigm focuses on qualitative research from political science, while spatial analysis from geography is relatively limited; the spatial perspective focuses on the basin or regional scale, while some global scale evaluations also mostly use basins as the analysis unit; and compared with qualitative research or policy review, quantitative research is less and lacks analysis from the perspective of social networks.

With the state as the basic unit of analysis, this article attempts to explore the cooperative relationships behind the global TWC events in the Post-Cold War era from the perspective of space, network, and relations. Complementing related studies, this article focuses on more general answers to several key questions: what kind of spatial linkages feature in global TWC, and what role do some key state actors play in it? With the profound evolution of globalism and interdependence, to what extent does the relationship between countries have an impact on TWC, and what mechanism does the process contain?

#### 2.1.2. Multidimensional Proximity

Seeking to relate spatial analysis with the analysis of international relations to address these questions is particularly complex. Similar to geographical research, many international relations issues also emphasize the importance of multidimensional factors analysis, such as the understanding of history, geography, politics, economy, diplomacy, and factors related to religion [49]. The essence of TWC is the concrete manifestation of the relationship between countries in a specific practical activity. The production and deepening of this activity are bound to be affected by the comprehensive influence of political, economic, and cultural relations between countries. Therefore, it is feasible to apply the multidimensional perspective of geographical research [50] to the study of TWC relations, which has practical significance for crossing the current paradigm barriers in this field and making up for the lack of a single analytical perspective.

Multidimensional proximity provides an effective research framework for this attempt. Proximity is a concept widely used in geography and regional science [51]. The perspective for observing proximity is usually divided into monadic and dyadic. Proximity at the monadic level refers to the spatial proximity or coverage of a region, which emphasizes a phenomenon of spatial agglomeration. Proximity at the dyadic level focuses on the proximity between two regions or individuals, which emphasizes the distance (or differences and similarities) between two interactive regions or individuals. The latter is more commonly discussed in academic research. Proximity initially only refers to geographical proximity, that is, the distance between things [52]. It is generally considered to come from the observation of industrial agglomeration and knowledge spillover by economic geographers [53]. The spatial agglomeration of innovation activities makes people first realize the importance of geographical proximity to innovation. There is a substantial body of work on the relationship between geography and innovation, which explains that close geographical distance between actors is more conducive to face-to-face communication and interaction [54,55,56]. Additionally, frequent exchanges help to create a good cooperative relationship between actors, thereby enhancing the circulation of tacit knowledge and the production of innovative activities [57].

As research progressed further, scholars found that single geographic proximity could not fully explain the generation of innovation. Therefore, the multidimensionality of the research perspective has received attention and discussion. The French school of proximity proposed that proximity should include multiple dimensions, and suggests that proximity in other dimensions also had an important impact on the research object [58]. In addition to physical proximity, socioeconomic interdependence should also be considered [51]. Boschma systematically defined the concepts of cognitive and organizational, while language, and cultural proximity were also widely mentioned by other researchers [59,60,61,62]. What these dimensions have in common is that being proximate in any of them enhances coordination and reduces uncertainty, thereby contributing to knowledge production and innovation [53,60].

The flourishing of the theory of multidimensional proximity has resonated with other disciplines and broken through its early spatial scale perspective that focused only on local activities. On the one hand, a major research direction focuses on the geographical constraints of network formation and its evolution. This approach is based on the findings that geographic proximity tends to facilitate the formation of networks, which increases social contact, information exchange, and the creation of social relationships [57]. This also influences some scholars to classify these explanations as part of the geographical theories of networks [63]. Furthermore, criticism of the early concept of localized networks pointed out that such networks may not bring the expected effects of innovation, and “spatial myopia” or “lock-in” would reduce the explanation of localized networks [60,64,65]. In contrast, global relations based on economic globalization and the adjustment of production relations revealed that this theory can be applied to a wider range of spatial scale studies, such as global production networks or global value chains [66,67]. On the other hand, multidimensional proximity is recognized in other disciplines or studies that emphasize relational and geographical perspectives, and one of the important areas is international relations. Using geopolitics as a link, some scholars have provided linkages between the disciplines of political science and geography through the integration of international relations and political geography [68]. For example, Harvey Starr [68] advocated proximity in his research on international conflict, emphasizing the importance of geography, distance, and spatiality in theoretical and empirical work on international relations. Some scholars have also pointed out that proximity is a strong factor in predicting dyadic conflict, and the greater the “distance” between states, the greater the probability of conflict [69]. Although multidimensional proximity has been applied in many research fields, for transboundary water cooperation, there is still a lack of discussion on its generative mechanism from this perspective.

### 2.2. Theoretical Framework: Towards Global TWC

**Figure 1:**
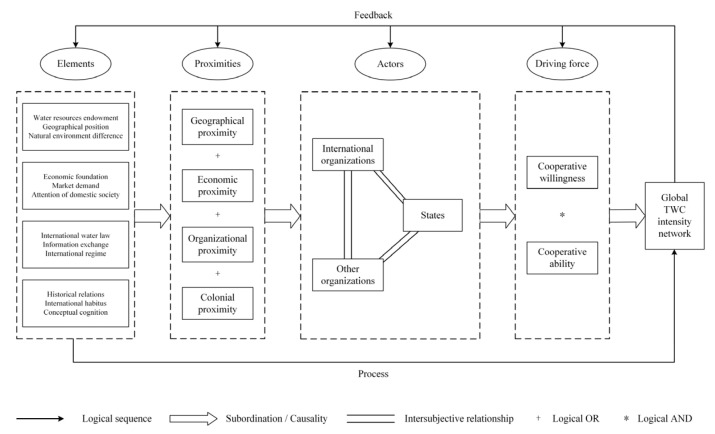
The theoretical framework for the formation of global TWC.

In our analysis, we conduct macro-level research on TWC from the perspective of inter-country relations and select four different proximity forms of geographical, economic, organizational, and colonial, to analyze the general mechanism which affects the intensity of global TWC (Figure 1).

#### 2.2.1. The Relations of Proximities and Global TWC

Geographical proximity. The influence mechanism of geographical proximity on TWC is mainly manifested in three aspects: water resources endowment, geographical position, and natural environment difference between state actors. Countries with favorable water resources endowment tend to have weaker willingness to cooperate, such as countries with a high water supply and low water demand. Conversely, countries with a low water supply and high water demand will show a high willingness to cooperate. In terms of geographic position, as Tobler’s [70] first law of geography revealed, the distance between things in space is inversely proportional to the closeness of relationships, and the closer geographical distance between countries or the direct existence of basin links is more conducive to communication and interaction. Besides the factor of geographical distance, the actor in the same transboundary basin should be taken into account. Geographical proximity will also affect countries’ understanding of the environment and further affect their diplomatic behavior, prompting them to formulate foreign policies consistent with the environment [71].

Economic proximity. The effect of economic proximity on TWC is mainly manifested in the economic foundation, market demand, and attention of domestic society. The economic foundation is the basis of each actor’s behavioral ability. Although some countries have a high willingness to cooperate, their economic development level is relatively weak, and they are unable to undertake international responsibilities or take effective actions in cooperation, which restricts the development of TWC between themselves and other countries or international organizations. Market demand emphasizes the attraction of other countries’ water markets to one country. Positive demand, negative demand, and potential demand have different degrees of influence on the country’s TWC cooperative willingness and ability. Trade flows and trade-based interdependence among countries also contribute to the promotion of peace and cooperation among countries [72]. The domestic social environment, interest groups, and other factors will affect national policymakers in formulating foreign policy [73]. Furthermore, the pursuit of seeking economic benefits on TWC drives interest groups to lobby the government to water cooperation [74].

Organizational proximity. As Boschma pointed out, organizational proximity includes the similarities that participants are connected by sharing the same reference space and knowledge [60]. Additionally, it often means the same space of relations based on the effective interactions of various nature [75]. It includes a relation of similarity and a relation of membership. Organizational proximity mainly affects the formation of TWC from three aspects: international water law, information exchange, and international regime. International water law refers to a series of treaties or practices reached among the international actors to solve international water resources development and protection. Strictly speaking, there is currently no unified international water law [76]. However, international water law emphasizes the goal of equitable development and sustainable use of transboundary water resources, regulates the rights and obligations of international actors, and helps to safeguard and enhance the cooperative ability of all actors. The information exchange helps to ensure the effectiveness of communication among the actors in the organization. The higher the level of information exchange and the more formalized and institutionalized the process, the more conducive to the smooth flow of tacit knowledge, which could influence the actors to reach a consensus on cooperation. The international regime is a series of principles, norms, rules, and decision-making procedures formed around the expectations of actors in a given field of international relations. Neoliberal institutionalists, such as Keohane, emphasized that an effective regime can promote official contacts and establish cross-governmental communication networks [77]. The more international regimes established between countries means that they not only have more options for cooperation channels in dealing with specific issues, but can also avoid the possibility of losing cooperative participation due to the failure of a single rule.

Colonial proximity. Colonial proximity also affects the formation of TWC mainly from three aspects: historical relations, international habitus, and conceptual cognition. Historically, the close relationship between the colonizers and the colonized was derived from the decolonization policy adopted by the colonizers after World War II, which emphasized that the actions taken by colonial countries in the process of the collapse of the colonial empire were aimed at maximizing their interests. Compared with historical relations, international habitus emphasizes the current behavioral tendencies of the countries. The habitus of the actor derives from its long-term practice, is acquired through lasting experience in its social status, and is the practical logic of its action [78]. Although in the post-colonial system, countries have gained sovereign independence and equality in the sense of international law [79], under the influence of international habitus, the former colonial powers still actively dominate international affairs and reshape the international order by their strength. Additionally, the former colonies, influenced deeply by their politics, economy, as well as culture, not only maintain direct contact and cooperation with the former colonial powers in many affairs, but also have been indirectly affected in the practice of cooperation with other countries. Conceptual cognition is another factor that affects TWC between former colonial powers and colonies, as well as between colonies. Lu [80] pointed out that some former colonial powers, out of compensation for their historical responsibilities, strengthened rectification justice or political reconciliation in their interactions with former colonies, so as to carry out cooperative activities with a nature of assistance in many fields, including water cooperation. From the perspective of constructivism, since countries that have been colonized by the same colonial power may have similar language, norms, and culture, they can help countries to shape identity [72]. Therefore, these countries can form more effective communication and promote water cooperation activities in dealing with water affairs.

#### 2.2.2. Actors and Driving Force

Global TWC is the result of conscious and purposeful interaction between actors. Under the influence of multidimensional proximity, the cooperative subjects jointly promote the deepening of water cooperation. This process has shaped the spatial patterns of TWC in the Post-Cold War era. The formation and deepening of TWC is usually a bilateral or even multilateral process. Cooperation among and within various actors, including the states, international organizations, and other organizations, shapes the pattern of global TWC. Among them, we argue that the states are the most important actors, which have a rational behavior, and their participation in global governance is based on the analysis logic of “cost-benefit”. International organizations are also the basic unit of global governance and have independent status in participating in international affairs, but they have a certain degree of “idealism” in their goals and behaviors, so they are slightly less rational. Compared with state actors and international organizations, other organizations or agencies are usually affiliated with states and have limited participation in water cooperation. For example, private actors represented by companies or corporations are actually representatives or executors of the will and decision-making of the states in TWC affairs, and the TWC issue will ultimately be resolved at the national, regional, or international level. Therefore, we argue that the success of TWC depends on the willingness and ability of state actors to cooperate in this field.

The cooperative willingness is the inclination or preference shown by the actor after a comprehensive analysis of the benefits and costs in achieving the goal of making cooperative commitments or fulfilling cooperative obligations in TWC affairs; and the cooperative ability is the actual conditions and level of the actor in undertaking costs in order to obtain benefits. When actors cooperate in transboundary water issues, their willingness and ability to constitute the independent variables of the result together, and the combination of the two constitutes sufficient conditions for this result. Thus, ability without willingness, or vice-versa, is logically and practically insufficient to produce TWC behavior. For example, the reason why China and the EU can play a leading role in regional TWC is closely related to their high willingness and ability to cooperate; while many emerging countries have a high willingness to participate in TWC, their limited cooperation ability makes it impossible to achieve effective water cooperation with relevant actors. Cooperative willingness and cooperative ability drive the establishment and formation of TWC among and within state and non-state actors, but both of these are affected by multidimensional proximity. Geographical, economic, organizational, and colonial proximity are sufficient conditions for the willingness and ability of the actors to cooperate. That is, proximities not only act individually on TWC, but also promote the development of the actor’s cooperative willingness and cooperative ability through appropriate combinations, which could further have a positive effect on the formation and intensity of TWC.

## 3. Data and Methods

### 3.1. Research Area

**Figure 2:**
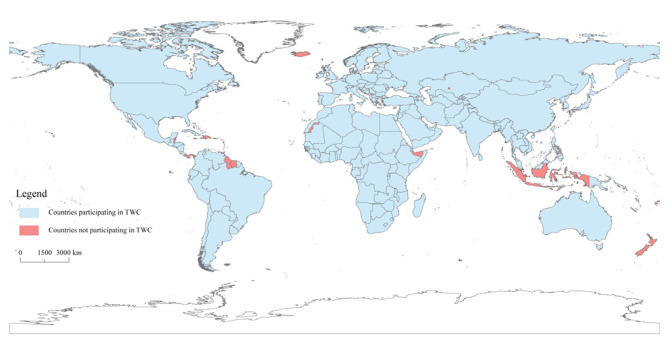
Spatial distribution of countries participating in TWC.

As more and more countries participate in the TWC, the geographic coverage of TWC events spans the globe. The main analysis of the article focuses on water cooperation activities in the Post-Cold War era, mainly involving 152 countries. Except for historical countries including Czechoslovakia, SFR Yugoslavia, FR Yugoslavia, as well as Serbia and Montenegro, other countries are shown in Figure 2. These countries are divided by continents: Asia (45 countries), Africa (45 countries), Europe (35 countries), North America (11 countries), South America (10 countries), and Oceania (two countries).

### 3.2. Data

#### 3.2.1. Water Cooperation Events Data

Interaction events reflect the relationship between countries, and events data analysis is a basic method to quantitatively measure bilateral relations [81]. The research object of this article is global TWC, therefore, the basic analysis unit is global TWC events. Data on global TWC and conflict events from 1948 to 2008 have been collected by the International Water Event Database (IWED) of Oregon State University [46,48,82], while establishing a coding system based on the nature and intensity of events. The nature of the event is divided into three categories, and the intensity is assigned to 15 levels, which represent negative water events (intensity level from −7 to −1), neutral water events (intensity is 0), and positive water events (intensity level from 1 to 7) (Table A1). The absolute value of the level is proportional to the intensity of the event. The database provides a solid grounding for the success of earlier TWC research [2,45,83].

However, the database has not been further updated since its release, and the pattern and law of TWC in the past 10 years need to be described urgently. According to the IWED data retrieval rules, we retrieved news event materials related to freshwater resources from 2009 to 2013 through the World News Connection (WNC) database, and a total of 80,783 news events were obtained. After conducting two different levels of data filtering and coding, 303 TWC events were finally confirmed.

Based on the above process, the article obtained a total of 1423 TWC events, including 4756 cooperation linkages. The data covered 22 years (from 1992 to 2013) and 152 countries (including historical countries).

#### 3.2.2. Multidimensional Proximity Data

The dependent variable measured by the multidimensional proximity model in this article is the total intensity of TWC between state actors from 1992 to 2013. To avoid statistical differences caused by territorial changes, state actors do not include historical countries and the newly independent country South Sudan, which ultimately contains 147 national actors.

For the independent variables, the study selects six specific indicators to quantify the proximities among countries, including the distance between capitals of countries, whether both countries are members of the same one international organization involved in TWC, their bilateral trade volume, whether they have the same water organization, whether they had a colonial link, whether they had a common colonizer (Table 1). These indicators are used to reflect the four proximities: geographical, economic, organizational, and colonial proximity.

Based on the above process, the study established a multidimensional proximity database of global TWC, including one dependent variable matrix and six independent variable matrices, with a total of 151,263 analysis units. The data covered 22 years (from 1992 to 2013) and 147 countries.

### 3.3. Methods

#### 3.3.1. Social Network Analysis

A social network is a collection of social actors as nodes and their relationships. For international transboundary water cooperation, it can be abstracted as a network collection with state actors as nodes and cooperative linkages as social ties. Based on the research needs, this article eventually established the undirected weighted network:

(1) C=(N,R),

where C is the global TWC network; N is the nodes of state actors; and R is the water cooperation linkages weighted by connection frequency or connection intensity.

The centrality of a node reflects its influence in the network. According to the theory of social network analysis, the degree, weighted degree centrality, and weighted betweenness centrality [84] are introduced to analyze the individual network characteristics of the TWC network, so as to quantify the importance and connectivity of the nodes (Table 2).

#### 3.3.2. QAP Analysis

The traditional multiple regression model is based on the ordinary least squares (OLS) method, and its basic assumption is that there is no correlation between the independent variables. In the real world, however, “relationships” are usually not independent. To investigate the determinants of TWC, quadratic assignment procedure (QAP) is used.

QAP analysis is a nonparametric test applied to the “relationship-relationship” level. Its purpose is to examine the regression relationship between a matrix and other multiple matrices, as well as to evaluate the influence and significance of each independent variable on the dependent variable. Different from the OLS regression model, QAP regression does not require assumptions on the mutual independence between variables. In the QAP procedure for network analysis, the standard errors are estimated using repeated permutations of the data set [85]. The calculating logic of QAP is consistent with the analytical logic of multidimensional proximity, and the essence is to explore the degree of “proximity” between actors from the perspective of the relationship. Therefore, the QAP regression model is established as follows:

(2) $$Y=\beta_{0}+\beta_{1}X_{1}+\beta_{2}X_{2}+\ldots +\beta_{n}X_{n}+\mu ,$$

where Y is the dependent variable matrix, depicting the intensity of TWC between countries; and $X_{1}$, $X_{2}$,…, $X_{n}$ as the independent variable matrices, which are specific indicators of multidimensional proximity between countries.

## 4. Results

### 4.1. Time Series of TWC Events

**Figure 3:**
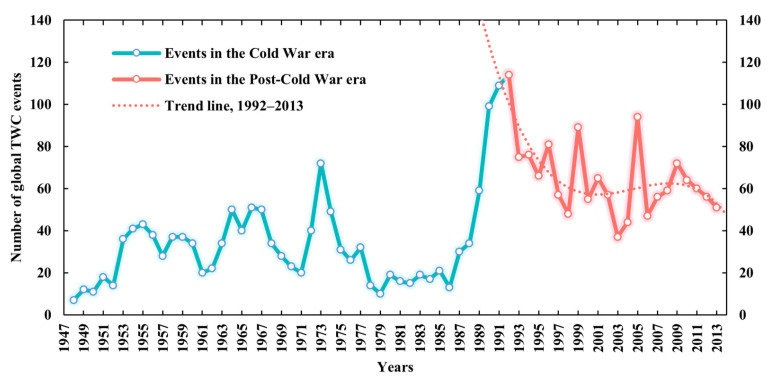
Annual variations of the number of global TWC events.

The latest update allows us to analyze the TWC trends more precisely. To capture their dynamics, the article counted the number of global TWC events by year, as shown in Figure 3. The latest TWC time series shows that there were 1423 water cooperation events around the world from 1948 to 2013. The maximum number of events appeared in 1992, which was 114; the minimum appeared in 1948, which was only seven events. The overall scale of events also increased significantly, from 33 in the Cold War era to 64.7 in the Post-Cold War era on the annual average level. In a certain period of time, the changes in the number of events were often not linear, mostly fluctuating. It can be found that sharp changes occurred around 1991, the number of events rise from 13 in 1986 to 114 in 1992.

The key reasons for these characteristics are the disintegration of the bipolar system and the development of the multi-polarization trend, namely, the change of the international system. From 1989 to 1991, major geopolitical events occurred in succession within three years. In particular, on 25 December 1991, the Soviet Union formally collapsed into 15 countries, resulting in an increase in the number of transboundary rivers and basins, as well as TWC events. After that, in the Post-Cold War era, peace and development became the themes of the times. With the ease in international political tension, the scale of TWC in this stage is higher than that of the Cold War era, and the interaction between countries has shown steady fluctuations.

### 4.2. Spatial Differentiation of TWC Linkages Based on Frequency

Taking state actors as nodes, TWC linkages in the Post-Cold War era as edges, and applying the connection frequency to give weight, a global TWC frequency network $C_{1}$ is constructed. The weighted degree centrality and weighted betweenness centrality of nodes in the network are calculated and Table 3 shows the top countries ranked by them. In terms of weighted degree centrality, the top 20 countries are all from Europe-North America, Africa, and Asia, and their distribution is relatively balanced, with eight, seven, and five countries, respectively. Compared with the former, the ranking of weighted betweenness centrality differs more among regions. Europe-North America, Africa, and Asia have five, five, and 10 countries, respectively, and more than half of countries come from Asia. Specifically, China, Egypt, Germany, the United States, and Russia have always occupied the top five in the two indicators, with China always occupying the first place. On the basis that they have the cooperative ability, this result is mainly related to the geographic and environmental factors of these countries. These countries have longer border lengths or a larger number of neighboring countries, which naturally determines their needs and willingness for TWC. However, it can also be found that for some countries with short borders and few neighboring countries, their status in the network is also prominent. The reasonable explanation is that this is related to their own specific interest demands, which include both water-related and non-water-related interests. Some countries have high water security needs, so they would actively take TWC to meet their water-related interests, such as Israel. Other countries are more expected to meet other interests through TWC, such as questing for their international status or enhancing their national image. A typical case is Japan. After World War II, Japan has long carried out economic diplomacy with ODA (Official Development Assistance) as the main means and provided assistance to many countries, and TWC affairs are one of its priorities. Therefore, while exporting its own successful water management experience, Japan continuously expands its political and economic interests as well as enhances its international image.

**Figure 4:**
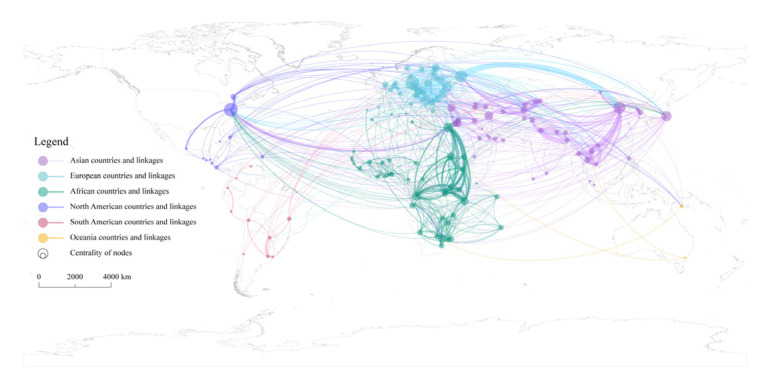
Spatial pattern of transboundary water cooperation linkages based on frequency weighting.

To make better sense of the network structure, the spatial pattern of TWC between countries is illustrated. As shown in Figure 4, the frequency network of TWC in the Post-Cold War era has obvious topological and spatial structure heterogeneity. First, Asian countries participate in TWC much more frequently than others, and the local structure of the network in Asia is also denser and more complex. The highest frequency of cooperation has occurred between China and Russia, up to 78 times. Among the top 20 partnerships, there are 14.5 pairs of Asian countries. Second, the network structure consists of triangular or quadrilateral structures within the continent, which are commonly found in the Nile, Zambezi River Basin in Africa, Danube River Basin in Europe, Mekong River Basin in Asia, etc. Clearly this shows that TWC is sensitive to geographical distance, and its geographical proximity is prominent. Third, extra-regional powers are widely involved in TWC. On the one hand, intercontinental interaction among countries is obvious, for example, the United States and Canada are widely involved in TWC in Asia, while European countries maintain a high level of interaction with African and South American countries. On the other hand, some island countries actively participate in TWC among continental countries. For example, Japan has extensive cooperation with countries in East, Southeast, and West Asia. Additionally, the UK has extensively established cooperative relations with countries in East and West Africa.

### 4.3. Spatial Differentiation of TWC Linkages Based on Intensity

Cooperation frequency can reflect the scale of cooperation, but cooperation intensity can more effectively reflect the quality of cooperation. Taking state actors as nodes, TWC linkages in the Post-Cold War era as edges, and applying the connection intensity to give weight, a global TWC intensity network $C_{2}$ is constructed. Calculating the weighted degree centrality and weighted betweenness centrality of state actors (Table 3), on the one hand, it can be found that among the top 20 countries compared with network $C_{1}$, the proportion of Asian countries has remained stable, and the proportion of European countries has increased. Most of the countries with high centrality are located in the surrounding areas of China, as well as Eastern and Southern Europe. On the other hand, the status of extra-regional countries, such as the United States and Japan, has declined.

For the former, its cause is inseparable from the constraints of the geographical environment and the relatively successful mechanism construction of the areas. In Asia, as Asia’s water tower, the Tibetan Plateau closely connects China and neighboring countries through transboundary rivers, making the region have a lot of water cooperation needs and practices. In Europe, due to the high level of regional integration and the relatively complete construction of cooperation mechanisms, countries usually carry out high-intensity water cooperation.

For the latter, the cause may be that the cooperative willingness of countries outside the region is weaker than that inside the region. Although countries such as the United States, Japan, and South Korea have a prominent centrality in the frequency network, they are not located in the hot spot basins, and their participation in TWC is mostly in the form of economic and technical assistance. Therefore, they are less likely to achieve in-depth and decisive cooperation results with relevant countries than local participants. It is worth noting that China’s two centrality indicators both rank first in both frequency and intensity networks, reflecting that China occupies an extremely important position in the network and is a very important participant in global TWC.

**Figure 5:**
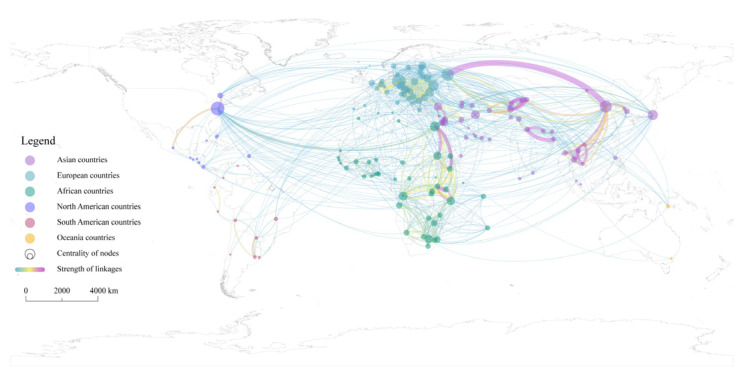
Spatial pattern of transboundary water cooperation linkages based on intensity weighting.

In terms of network linkages, it can be found that the topological and spatial structure heterogeneity of the TWC intensity network has become more obvious compared with the frequency network (Figure 5). First, the network hierarchy is obvious and there are far more low strength linkages than high strength linkages. Linkages with a strength higher than 10 accounted for only 36.1% of the total. Second, Asia is the continent with the most complex TWC spatial pattern and the highest concentration of hot spots. High-intensity water cooperation runs through the Eurasian and African continents. High strength linkages only exist between geographically neighboring countries within a certain geographic area. Countries located in the Amur, Mekong, Ganges, Indian, Aral Sea, Jordan, and the Nile River Basin have carried out high-intensity water cooperation.

### 4.4. QAP Multiple Regression Results

By importing the multidimensional proximity variable matrices of global TWC into the QAP regression model, and then having performed 2000 times matrix random permutations to estimate the standard errors, the regression results were obtained. Table 4 reports the results of the QAP regression. The goodness of model fit is 0.272, indicating that the variables can explain the difference in TWC intensity between countries. The regression results show that geographical proximity, economic proximity, organizational proximity, and colonial proximity have significant effects on TWC. This also allows our theoretical framework to be quantitatively verified.

First, the significant impact of geographical proximity on TWC has been fully verified. On the one hand, the capital distance between countries is negatively correlated with the intensity of TWC, and the result is significant at the 0.1% level, indicating that the closer the countries are, the greater the likelihood and intensity of TWC. On the other hand, whether countries belong to the same transboundary basin is positively correlated with the intensity of TWC. The result is significant at the 0.1% level, indicating that high-intensity TWC is more likely to occur between countries with spatial connections at the transboundary basins. Additionally, this result is also clearly reflected in the spatial pattern of the TWC intensity network (Figure 5). Second, economic proximity has a significant positive effect on TWC. The bilateral trade volume, as its specific indicator, is significant at the 0.1% level, indicating that closer trade between countries is more conducive to the development and deepening of TWC. With the development of globalization, the dependence of economy and trade have increasingly become the anchor of political relations between countries [86]. The higher the degree of trade dependence between countries, the more it leads to shared benefits, which in turn will affect political relations between countries and promote mutual cooperation. Third, organizational proximity has a significant positive effect on TWC, and the result is significant at the 0.1% level, which means that the more water organizations exist among countries, the greater the intensity of TWC. For example, a variety of cooperation regimes have been formed in the Mekong River Basin [87], including the GMS (Greater Mekong Subregion Economic Cooperation), the MRC (Mekong River Commission), the AMBDC (ASEAN-Mekong Basin Development Cooperation), the LMI (Lower Mekong Initiative), the MGCI (Mekong-Ganga Cooperation Initiative), and the LMC (Lancang-Mekong Cooperation). These regimes provide various dialogue platforms for the basin countries and play an important role in promoting transboundary water governance and economic cooperation in the region. Fourth, colonial proximity is significantly positively correlated with the intensity of TWC, and the results of each indicator are significant at the 0.1% level. This suggests that former colonies prefer to maintain a high level of cooperation in transboundary water matters with former colonizers as well as other former colonies. For example, in 2007, Uganda and the Congo-Kinshasa had to refer to the agreements and maps reached in Europe in the past to resolve the dispute over the lake islands between the two countries. Another example is when the UK established a new close bond by transferring power to the regime that was most beneficial to its own interests while recognizing the independence of the colonies, and at the same time incorporated the newly independent country into the Commonwealth, thus establishing a new kind of close ties. Thus, in Figure 4 and Figure 5, we can see that the UK is widely involved in African water affairs.

## 5. Conclusions

Today, globalization and networking characteristics of international collaboration activities are particularly prominent. A systematic investigation of global TWC networks will substantially further our understanding in this field. Based on the mining of cooperation events data, the article builds the Post-Cold War era global TWC networks. Through the comprehensive use of social network analysis and QAP analysis methods, the topological structure and spatial pattern of TWC are revealed, and the multidimensional proximity mechanism of TWC is discussed. The analysis shows that:

First, in terms of the overall scale of TWC events, the annual change in the number of events was often not linear, mostly fluctuating. The number of events in 1992 was the peak over the years since 1948, and then the scale of events experienced a process of rapid decline and stable fluctuation. However, compared with the Cold War era, the overall scale of TWC events in the Post-Cold War era has increased significantly. The key reasons for these characteristics are the reconstruction of territorial space on the eve of the end of the Cold War and the change in the international system.

Second, in terms of the TWC network structure, the roles of different network actors are different, and the spatial heterogeneity of the TWC linkages is obvious. In the frequency network of TWC, the dominant countries are mostly distributed in Asia, Africa, Europe, and North America, but especially in Asia. China, Egypt, Germany, the United States, and Russia are the most important network nodes. Network ties are concentrated in the Eastern Hemisphere, especially the Eurasian continent and the African continent. Additionally, the extra-regional powers are widely involved in the TWCs of both the Eurasian and African continents. In the intensity network of TWC, the geographical proximity of the network has become more obvious, and the high strength linkages are further concentrated in a few regions. Countries located in the Amur, Mekong, Ganges, Indian, Aral Sea, Jordan, and the Nile River Basin have carried out high-intensity water cooperation.

Third, in terms of the proximity mechanism, TWC activities among state actors are not only affected by a single dimension of proximity, but by the comprehensive influence of multidimensional proximity. Overall, geographical proximity, economic proximity, organizational proximity, and colonial proximity significantly affect the intensity of water cooperation among countries. Specifically, the capital distance between countries is negatively correlated with the intensity of TWC. Whether countries belong to the same transboundary basin, the bilateral trade volume, the number of water organizations existing among countries, whether there is a colonial relationship between countries, and whether there is a common colonizer have significant positive effects on the intensity of TWC.

Fourth, spatial and regression analysis examined our theoretical framework for the influence of different dimensions of proximities on the generation of global TWC. This framework discusses the general process and mechanism of global TWC from the perspective of proximity, as well as the complex interaction and causal mechanisms. State actors are the main actors involved in global TWC, and close interaction and cooperation are carried out among and within the various actors. The formation and deepening of TWC depend on the willingness and ability of the actors. Both of them are indispensable and constitute sufficient conditions for TWC results. Cooperative willingness and ability are affected by multidimensional proximities, which are composed of various constituent elements. Each proximity can not only play an independent role, but also promote the development of the actor’s cooperative willingness and ability through appropriate combinations.

The global TWC network is a kind of complex and dynamic network. Based on the feasibility of data mining and cleaning, the time scale analyzed in this paper mainly covers the period from 1992 to 2013. It is still necessary to further update the data, especially since 2013, since with China’s proposal and implementation of the Belt and Road Initiative, the international cooperation pattern of Asia, Africa, and Europe is being profoundly reshaped. Therefore, although the current networks have shown the spatial pattern of in-depth combination with the core region of the Belt and Road Initiative, it is of positive significance to further research the new characteristics of TWC under the background of the Belt and Road Initiative. In addition, with the strengthening of interdependence and globalism, the deeper economic ties between countries are increasingly shaping both social and environmental ties, and the systematic correlation between different networks will become deeper. Therefore, further strengthening the research on the effects of linkage between TWC networks and other networks, such as energy trading network and food trading network, will help to understand the systemic effects and global governance underlying the background of globalization.

## Figures and Tables

**Table 1:** Definition of proximity indicators and their data sources.

Proximity Name	Indicator Name	Abbreviation	Definition	Source	Years
Geographical proximity	Geographical distance	dist	The geographical distance between the capitals of the two countries and standardize it	CEPII	2019
	Same transboundary basin	basin	Dummy variable that is 1 if two countries belong to the same transboundary basin, and 0 otherwise	IWED	2019
Economic proximity	Bilateral trade volume	trade	The cumulative value of bilateral trade volume between the two countries and standardize it	UNCTAD	1992–2013
Organizational proximity	Water organization	organ	The frequency that the two countries are in the same water organization	IWED	1948–2013
Colonial proximity	Colonial link	colony	Dummy variable that is 1 if two countries had a colonial relationship after 1945, and 0 otherwise	CEPII	1945–2019
	Common colonizer	comcol	Dummy variable that is 1 if two countries had a common colonizer after 1945, and 0 otherwise	CEPII	1945–2019

Notes: Water organization is an international organization that participates in TWC and currently exists. When the actors in a certain water event involve at least one international organization and it effectively plays a cooperative role, we regard it as a “water organization”. The statistics include not only its member states but also its observer states or dialogue partners.

**Table 2:** Main analysis indicators of network characteristics.

Indicator	Formula	Definition	Implication
Degree	$k_{i}$	Number of nodes directly connected to node i	The extent to which the actor is at the center of the network
Weighted degree centrality	$WC_{RD}\left(i\right)=\frac{C_{RD}\left(i\right)\sum_{i=1}^{n}w_{ij}}{{\left(\sum_{i=1}^{n}w_{ij}\right)}^{max}}$	The ratio of the number of nodes directly connected to node i to the maximum number of nodes that it may be directly connected. Weighted by the connection strength between nodes	The extent to which the actor is at the center of the network
Weighted betweenness centrality	$WC_{RB}\left(i\right)=\frac{C_{RB}\left(i\right)\sum_{i=1}^{n}w_{ij}}{{\left(\sum_{i=1}^{n}w_{ij}\right)}^{max}}$	The standardized value of the probability that node i is on the shortest path between node j and node k. Weighted by the connection strength between nodes	The extent to which the actor controls the contacts between other actors

Notes: Where n is the number of nodes in the network, $b_{jk}\left(i\right)$ is the probability that node i is on the shortest path between node j and node k, $w_{ij}$ is the connection strength between node i and j.

**Table 3:** Countries’ hierarchies based on weighted centrality indicators.

	$C_{1}$				$C_{2}$			
Rank	Country	Weighted Degree Centrality	Country	Weighted Betweenness Centrality	Country	Weighted Degree Centrality	Country	Weighted Betweenness Centrality
1	China	0.279	China	0.153	China	0.279	China	0.153
2	Germany	0.177	Egypt	0.052	Germany	0.240	Germany	0.068
3	Russia	0.171	Germany	0.050	Russia	0.200	Russia	0.050
4	Egypt	0.156	USA	0.045	Ukraine	0.138	USA	0.044
5	USA	0.121	Russia	0.043	Egypt	0.125	Egypt	0.042
6	Ukraine	0.111	Sudan	0.018	Tanzania	0.123	South Africa	0.022
7	Tanzania	0.106	Iran	0.017	USA	0.119	Iran	0.017
8	Congo-Kinshasa	0.095	Congo-Kinshasa	0.014	Congo-Kinshasa	0.091	Sudan	0.016
9	Sudan	0.088	Japan	0.013	Moldova	0.091	Congo-Kinshasa	0.014
10	Moldova	0.080	Jordan	0.013	Sudan	0.077	Tanzania	0.013
11	Thailand	0.071	Turkey	0.013	Tajikistan	0.077	Japan	0.012
12	Turkey	0.067	Tanzania	0.011	Czech	0.076	Turkey	0.011
13	Ethiopia	0.067	South Africa	0.010	Romania	0.075	Jordan	0.011
14	Czech	0.067	Ukraine	0.007	South Africa	0.067	Mali	0.009
15	Romania	0.064	Israel	0.006	Thailand	0.064	Ukraine	0.009
16	Jordan	0.063	India	0.005	Bulgaria	0.064	Czech	0.006
17	Tajikistan	0.059	Czech	0.005	Kazakhstan	0.060	Poland	0.006
18	Bulgaria	0.057	Syria	0.005	Turkey	0.059	Zimbabwe	0.006
19	Kenya	0.056	South Korea	0.005	Ethiopia	0.056	Israel	0.005
20	Uganda	0.055	Laos	0.005	Hungary	0.055	Laos	0.005

**Table 4:** QAP multiple regression results.

Variable	Unstandardized Coefficient	Standardized Coefficient	*p*-Value	Standard Error
dist	−0.84635	−0.03839	0.0005	0.24382
basin	13.32802	0.47452	0.0005	0.20493
trade	21.97590	0.09077	0.001	2.11566
organ	0.07188	0.04437	0.0005	0.01968
colony	1.43859	0.03712	0.001	0.29427
comcol	0.95653	0.05921	0.0005	0.14187
Intercept	−1.00595	0	0	0
$R^{2}$	0.2722			
$\mathrm{Adjusted}\;R^{2}$	0.27199			

## Data Availability

Not applicable.

## Appendix A

**Table A1:** The intensity scale of TWC events [47].

Intensity Scale	Descriptions
−7	Formal declaration of war
−6	Extensive war acts causing deaths, dislocation or high strategic cost
−5	Small scale military acts
−4	Political-military hostile actions
−3	Diplomatic-economic hostile actions
−2	Strong verbal expressions displaying hostility in interaction
−1	Mild verbal expressions displaying discord in interaction
0	Neutral or non-significant acts for the inter-nation situation
1	Minor official exchanges, talks or policy expressions—mild verbal support
2	Official verbal support of goals, values, or regime
3	Cultural or scientific agreement or support (nonstrategic)
4	Non-military economic, technological or industrial agreement
5	Military economic or strategic support
6	International freshwater treaty; major strategic alliance (regional or international)
7	Voluntary unification into one nation
